# A thermostable messenger RNA based vaccine against rabies

**DOI:** 10.1371/journal.pntd.0006108

**Published:** 2017-12-07

**Authors:** Lothar Stitz, Annette Vogel, Margit Schnee, Daniel Voss, Susanne Rauch, Thorsten Mutzke, Thomas Ketterer, Thomas Kramps, Benjamin Petsch

**Affiliations:** 1 Friedrich-Loeffler-Institut, Greifswald-Insel Riems, Germany; 2 CureVac AG, Tübingen, Germany; Wistar Institute, UNITED STATES

## Abstract

Although effective rabies virus vaccines have been existing for decades, each year, rabies virus infections still cause around 50.000 fatalities worldwide. Most of these cases occur in developing countries, where these vaccines are not available. The reasons for this are the prohibitive high costs of cell culture or egg grown rabies virus vaccines and the lack of a functional cold chain in many regions in which rabies virus is endemic. Here, we describe the excellent temperature resistance of a non-replicating mRNA based rabies virus vaccine encoding the rabies virus glycoprotein (RABV-G). Prolonged storage of the vaccine from -80°C to up to +70°C for several months did not impact the protective capacity of the mRNA vaccine. Efficacy after storage was demonstrated by the induction of rabies specific virus neutralizing antibodies and protection in mice against lethal rabies infection. Moreover, storing the vaccine at oscillating temperatures between +4° and +56°C for 20 cycles in order to simulate interruptions of the cold chain during vaccine transport, did not affect the vaccine’s immunogenicity and protective characteristics, indicating that maintenance of a cold chain is not essential for this vaccine.

## Author summary

Conventional prophylactic vaccines require transport and storage under controlled temperatures in an unbroken cold chain. Therefore, distribution of many vaccines is restricted to areas where the cold chain can be maintained which excludes especially rural areas in many countries from continues vaccine supply. Unfortunately, some diseases that can be prevented by vaccination, like the rabies virus infection, are still endemic in such areas. Therefore, logistic reasons often prevent delivery of life saving vaccines to areas in which they are most needed. Here, we describe an mRNA vaccine encoding the rabies virus glycoprotein (RABV-G) that remains protective in a mouse challenge model upon storage at highly variable temperatures. These results suggest that such a vaccine allows storage outside the cold chain and can therefore reach all areas of the world where rabies virus is endemic. Since mRNA vaccines consist of the same biochemical components, irrespective of the encoded protein, it is reasonable to assume that the thermostability observed for the rabies vaccine is a general characteristic of mRNA based vaccines.

## Introduction

Vaccines are effective means for protecting exposed individuals against infectious diseases, thereby improving people’s quality of life and public health. Current vaccines require a reliable cold chain during transport and storage that is technically complex, costly, and potentially prone to disruption [1]. This limits the global use of already existing effective vaccines in many areas of the world. Vaccines may accidentally be exposed to freezing temperatures or to heat [2,3] either of which, depending on the nature of the vaccine, may reduce vaccine efficacy [2,4]. In general, the stability of different vaccines is given at temperatures between 2° to 8°C and between 25° to 37°C and can range in duration from weeks to up to 2 years [5]. While thermostable vaccines promise to improve access in subtropical and tropical climates and in settings with limited resources, development of today’s vaccines is greatly complicated by the need for laborious and product-specific optimization in order to facilitate thermostability. Thus *per se* thermostable vaccines would present a major step forward in medical use in general and in supplying regions with extreme climates in particular.

Here, we demonstrate that synthetic messenger RNA (mRNA) may serve as a technology platform for simplified generation of thermostable vaccines. RNA vaccines exhibit immunological versatility by inducing cellular and humoral immune responses, defined composition, and allow for a rapid and standardized manufacturing process. Recently, anti-infective effects have been demonstrated for synthetic RNA vaccines [6], including our reports on mRNA vaccines against influenza and rabies [7,8,12]. We previously showed induction of humoral and cell-mediated immune responses, efficacy for newborns and elderly and long-lasting immunity in animal models. Extending initial reports demonstrating RNA was stable under heat stress after lyophilization with trehalose [9] we have previously shown protective efficacy of an mRNA vaccine encoding influenza virus HA upon storage at 37°C for 3 weeks[8]. In the present report, we demonstrate stability of an mRNA based vaccine in a comprehensive study of thermostability in the stringent rabies virus challenge model. We used a proprietary mRNA vaccine technology (RNActive) that involves optimization of the mRNA sequence, formulation, and production at high purity, as described elsewhere [10,11]. To validate and extend our previous findings, an mRNA vaccine encoding the rabies virus glycoprotein (RABV-G) was used as an execution example of a prophylactic mRNA vaccine. The used mRNA vaccine was stored in lyophilized form and reconstituted in buffer before injection. Since thermostability refers to resilience to both low and high temperatures and is usually tested at 2–8°C, 25°C, 37°C and ≥45°C [1,5], we conducted a series of experiments to explore protective efficacy subsequent to both low and high temperature storage for up to twelve months. We show that the candidate mRNA vaccine encoding the rabies virus glycoprotein retains immunogenicity and protective effects upon exposure to temperatures as high as 70°C and prolonged storage for several months. Importantly, results of phase I first-in-human clinical trial with the mRNA based rabies vaccine (CV7201) tested in the study here for thermostability have demonstrated that 32 of 45 (71%) subjects given 80 or 160μg intradermally and 6 of 13 (46%) with 200 or 400μg intramuscularly via needle-free device injection induced VNTs of 0.5 IU/ml or more across dose levels and schedules [12].

## Methods

### Virus

The rabies virus CVS-11 was received from the archive of Lyssaviruses of the OIE Reference Laboratory for Rabies and WHO Collaborating Centre for Rabies Surveillance and Research, Friedrich-Loeffler-Institut, Insel Riems, Germany. CVS-11 was grown on baby hamster kidney cells (BHK-21 from the collection of Cell Lines in Veterinary Medicine, Friedrich-Loeffler-Institut, Isles of Riems, Germany) and used throughout the experiments. Virus culture and all infectious experiments in animals were performed at the Friedrich-Loeffler-Institute, Federal Research Institute for Animal Health (Isles of Riems, Germany).

### mRNA- and protein based vaccines

**Fig 1:**
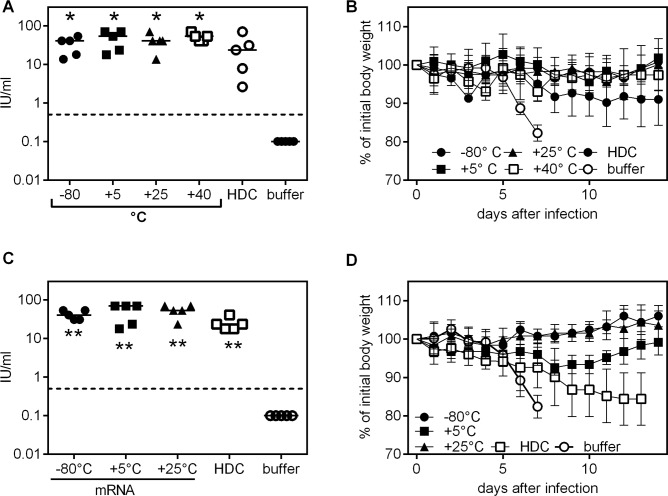
RABV-G mRNA vaccine stored at temperatures up to +40°C retains immunogenicity and protective for up to twelve months. (A-B: storage for six months). Vaccine or control injections were done on day 0 and 21. Induction of rabies virus neutralizing antibodies was measured on day 35 in mice vaccinated with RABV-G mRNA stored from -80°C to +40°C or HDC stored at the recommended temperature of 4°C for six months (dashed line in A and C indicates the WHO recommended titer of 0.5 international units per ml [IU/ml]). (C, D: storage for twelve months) Mice were challenged with 40fold MLD_50_ of rabies virus CVS-11, 71 (A, B) or 63 (C, D) days after the first immunization. Body weight was recorded after immunization using material stored for six (B) or (D) twelve months. For antibody titers, the mean is indicated, for body weight kinetics the mean and standard deviation is given (n = 5). Significance ** p < .001 to buffer control group was calculated using the Mann Whitney test.

The mRNA vaccine was based on CureVac's RNActive technology (claimed and described in patents EP1392341, EP1857122 and application WO2012019780A1) coding for the full-length rabies virus glycoprotein. mRNA vector structure and optimization was performed as described elsewhere containing a 5’ cap structure, 5’ UTR, open reading frame, 3’ UTR, and poly-A tail [7,13]. The complete nucleotide sequences and the complexation with the cationic peptide protamine of the used mRNA vaccines have been published before [7]. The licensed vaccines Rabipur (Novartis) and HDC (human diploid cell vaccine; Sanofi Pasteur MSD) are commercially available and were purchased from a local pharmacy. For thermostability testing, the mRNA vaccines in Fig 1 were stored at +5°±3°C (uncontrolled humidity), 25°±3°C (60% ± 5% rel. humidity) and 40°C ±3°C (75% ± 5% rel. humidity). mRNA in later trials, HDC and Rabipur were stored at indicated temperatures (uncontrolled humidity). Vaccines were stored as lyophilisates or reconstituted, respectively, as indicated. mRNA lyophilisates were reconstituted using ringer lactate solution prior to injection.

### Freeze-drying protocol

Borosilicate glass vials (type I) were half-closed with rubber stoppers prior to loading of the freeze-dryer. Vials were placed on the shelf of a state-of-the-art freeze- dryer (Lyoflex 04, BOC Edwards). The cycle included the lowering of the temperature to -40°C and freezing of the samples at this temperature for 2 h. The chamber pressure was reduced to 160 μbar, primary drying takes place at -10°C within 17 h, secondary drying was performed at 20°C for 10 h and 68 μbar. After lyophilization, vials were closed by lowering the upper shelf under nitrogen and sealed with an aluminum cap.

### Ethical statement

Six to eight weeks old inbred BALB/c were purchased from Janvier Laboratories (Le Genest-Saint-Isle, France). All animal experiments, including the rabies challenge infections using CVS-11,were conducted according to German laws and guidelines for animal protection and approved by the regional council Tübingen under reference numbers CUR 4/13.

### Immunization procedure

Mice were anesthetized by *intraperitoneal* application of ketamine (Sanofi-Aventis, Frankfurt, Germany) and Rompun (Bayer, Leverkusen, Germany). Thereafter, intradermal injection of 2 x 50 μl of ringer-lactate-solution alone or containing 80 μg (study in Fig 1) or 40μg of mRNA (subsequent studies) was performed using syringe and forceps for creating a skin fold. HDC and Rabipur were applied intramuscularly with 100 μl (0.1 human dose) distributed to four injection sites. All mice were immunized twice with a time interval of three weeks (day 0 and day 21).

### Antibody analysis

Blood samples were taken by retro-orbital bleeding. Anti-rabies serum antibodies were analyzed by FAVN test by Eurovir Hygiene-Institut, Luckenwalde, Germany according to WHO protocol [14]. All samples were anonymized prior to shipment to Eurovir.

### Challenge infection

After immunization procedure, animals were infected with rabies virus (for time interval between immunization and infection, see respective figure legend). The rabies challenge virus CVS-11 was applied by intracerebral (*i*.*c*.) injection with a volume of 20 μl and an infectious dose of 40 median lethal virus doses 50 (MLD50). Body weight and clinical signs of infected mice were assessed daily over a period of two weeks after infection. Mice with less than 80% of initial body weight or that were scored accordingly were sacrificed.

### Statistics

Statistical analysis was performed using GraphPad Prism software, Version 6.00. Statistical differences between groups were assessed by Mann Whitney test.

## Results/Discussion

In a first experiment, we tested the induction of virus neutralizing (VN) antibodies in BALB/c mice immunized with 80 μg of RABV-G encoding mRNA that had been stored at varying temperatures, ranging from -80°C to +40°C, for 6 months (Fig 1). As a control, a licensed inactivated rabies virus vaccine (HDC) that had been stored at 4°C as recommended by the manufacturer was used at 1/10 of the recommended human dose (100 μl), comparable to previously published work [15]. As shown in Fig 1A, the RABV-G mRNA vaccine induced protective RABV-G specific titers in the fluorescent antibody virus neutralization (FAVN) assay upon storage at -80°C for 6 months. Likewise, vaccination after storage of mRNA vaccine at 5°C and at slightly (25°C) or significantly (40°C) elevated temperatures induced high neutralizing antibody titers all significantly higher compared to the buffer treated control group (Fig 1A). Vaccinated mice were then challenged intracerebrally (*i*.*c*.) with 40-fold median lethal doses (LD_50_) of the rabies virus strain CVS-11. All immunized mice (mRNA and HDC controls) survived the challenge infection, whereas buffer treated control animals all had to be sacrificed at day eight due to drastic body weight loss of >20% (Fig 1B, S1 Table).

Next, we assessed the immunogenicity of vaccines stored at -80°, 5° or 25°C for 12 months in a challenge experiment in mice. The immunogenicity (Fig 1C) and protective capacity of the RABV-G mRNA vaccine was again not affected by storage. As before, none of the mRNA-immunized mice needed to be sacrificed upon *i*.*c*. challenge (S1 Table), although mice that received mRNA vaccine stored at 5°C showed a transient drop in body weight (Fig 1D). In contrast, two out of five mice immunized with the licensed vaccine HDC stored at 4°C for 12 months had to be sacrificed due to critical weight loss and one of the three surviving mice had a body weight only slightly above the critical threshold, indicating compromised vaccine efficacy for the HDC vaccine, potentially reflecting the reported lower potency of HDC compared to Rabipur [16].

**Fig 2:**
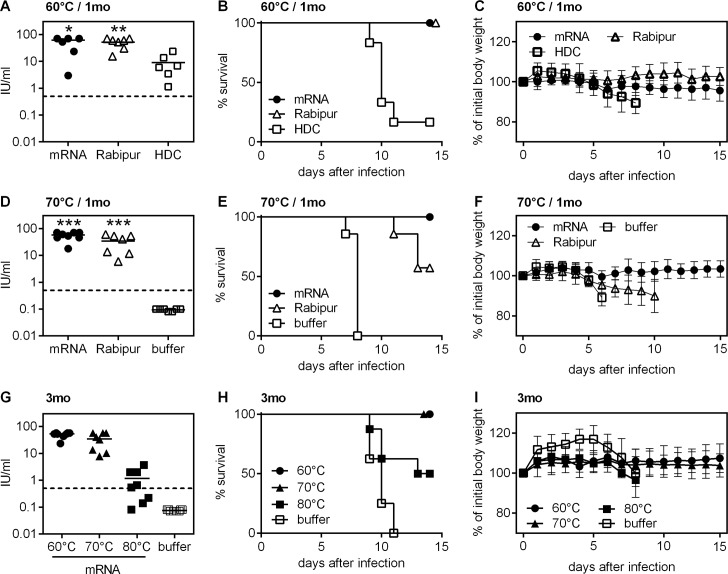
RABV-G mRNA vaccine stored at temperatures up to 70°C retains immunogenicity and protection for up to three months. mRNA vaccine or licensed vaccines were stored for one month (A-F) or three months (G-I). Storage temperature was 60°C (A-C), 70°C (D-F), or as indicated in the figure (G-I). Vaccine or control injections were done on day 0 and 21. Induction of rabies virus neutralizing antibodies was measured 35 days after first immunization (A, D, G; dashed line indicates the WHO standard titer of 0.5 IU/ml). Mice were challenged with a 40fold MLD_50_ of rabies virus CVS-11 on day 47 (B, C, E, F) and day 51 (H-I), respectively. Survival (B, E, H) and body weight (C, F, I) was recorded. For antibody titers, the mean is indicated, for body weight kinetics the mean and standard deviation is given (n = 6 to 8). Significance *p<0.1, **p<0.01, ***p < .001 to HDC or buffer control group in A and D/G, respectively, was calculated using the Mann Whitney test. Groups included in A-F were performed simultaneously; therefore buffer control displayed in D-F is valid for all groups.

We then further extended the temperature range by testing mRNA vaccine storage at 60°C. RABV-G mRNA and a second licensed inactivated rabies virus vaccine, Rabipur, both remained immunogenic (Fig 2A) and induced 100% survival upon rabies challenge if stored at 60°C for 4 weeks, unlike HDC that had lost its protective potency almost completely when stored under the same conditions (Fig 2B and 2C). In an extension of this experiment, we tested storage at 70°C for 1 month (Fig 2D–2F) and 60°C, 70°C and 80°C for 3 months (Fig 2G–2I). Mice that had been vaccinated with RABV-G mRNA stored at 70°C for 1 month were again completely protected against lethal challenge infection (Fig 2D–2F). In contrast, a licensed vaccine (Rabipur) only protected 60% of vaccinated mice against lethal infection (Fig 2E). Constant body weights of mice vaccinated with mRNA vaccines clearly reflect the robust protective efficacy of the vaccine after storage at this elevated temperature, while mice immunized with the control vaccine (Rabipur) showed a mean weight loss of up to 10% when the first mouse had to be sacrificed (Fig 2F). Since incubation at 70°C for 1 month did not result in a detectable decrease of immunogenicity and protective capacity of the mRNA vaccine, thermal stress was further increased to 3 months storage at 60°C, 70°C and 80°C. As shown in Fig 2G, 2H and 2I, immunogenicity was retained and protection was complete upon storage at 60°C and 70°C. However, at 80°C RABV-G mRNA lost some efficacy and was not completely protective, with 50% mortality upon challenge infection in this group (Fig 2H).

**Fig 3:**
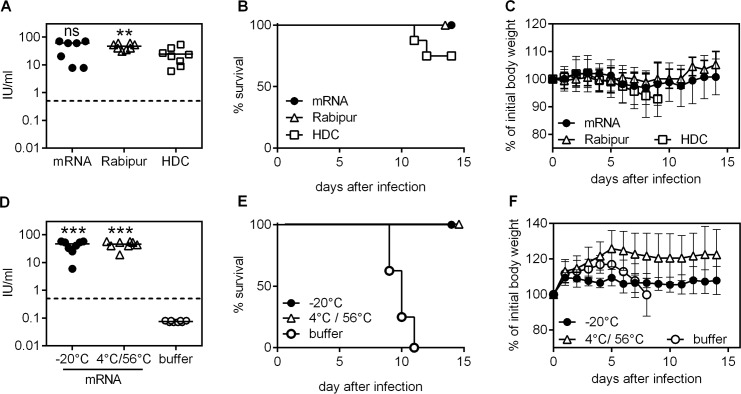
Oscillating temperatures and potential handling and storage errors tested do not compromise mRNA vaccine efficacy. mRNA vaccine, Rabipur and HDC were reconstituted in buffer and subsequently stored at +40°C for one week (A-C). In a second experiment, RABV-G mRNA was stored as a lyophilisate at 56°C for 8–9 hours per day and then at 4°C for 15–16 hours with a total of 20 cycles of oscillating temperatures (D-F). Vaccine or control injections were done on day 0 and 21. Induction of rabies virus neutralization titer was measured on day 35 after first immunization (A, D: dashed line indicates the WHO standard titer of 0.5 IU/ml). Mice were challenged with a 40fold MLD_50_ of rabies virus CVS-11 on day 47 (B-C) or day 51 (E-F) and survival (B, E) and body weight (C, F) was recorded. As groups in A-F were treated in parallel, the buffer control group in Fig 3D–3F is valid also for Fig 3A–3C. For antibody titers, the mean is indicated, for body weight kinetics the mean and standard deviation is shown (n = 6 to 8). Significance: ns = non-significant, **p<0.01, ***p < .001 to HDC or buffer control group in A and D, respectively, was calculated using the Mann Whitney test.

Finally, we conducted experiments to mimic handling and storage errors before application. First, we tested the stability of mRNA vaccines at 40°C for one week upon reconstitution in buffer and compared to the licensed benchmarks Rabipur and HDC. As shown in Fig 3A all RABV-G mRNA (40 μg/mouse) and Rabipur-vaccinated mice exhibited high titers of virus neutralizing antibodies and survived the challenge infection (Fig 3B) without weight loss (Fig 3C), whereas only 75% of mice in the HDC-vaccinated group survived (Fig 3B). Second, we assessed the immunogenicity of mRNA vaccine stored at changing temperatures alternating between 4°C and 56°C for one month (twenty cycles of 56°C for 8–9 h and 4°C for 15–16 h) in comparison to vaccines stored at -20°C. Induction of VN titers (Fig 3D) and survival upon challenge infection (Fig 3E) without weight loss (Fig 3F) clearly demonstrated that storage at alternating temperatures did not compromise the efficacy of the mRNA vaccine.

In this study, we demonstrated thermostability of a rabies-specific mRNA vaccine in the stringent *i*.*c*. rabies virus challenge model. To systematically assess thermostability of mRNA vaccines, we tested immunogenicity of a RABV-G mRNA vaccine stored at low (-80°C, 5°C) and at elevated temperatures (25°, 40°, 60°, 70°, 80°C) (S1 Table).

Since thermal stress is a function of both absolute temperature and duration of exposure, various exposure times were tested (three, six and twelve months). Under all conditions, except for extreme heat of 80°C for 3 months, tested, the RABV-G mRNA vaccine reliably induced high levels of VN antibodies (≥0.5 IU/ml), and also allowed the survival of immunized mice after lethal challenge with rabies virus. In contrast, licensed inactivated rabies virus vaccines used as benchmarks at least partially lost their protective capacity under the same experimental conditions.

As outlined above, most licensed vaccines are stable at temperatures between 2 and 8°C, which includes lyophilisates such as the yellow fever vaccine 17D. In contrast, data presented here provide evidence for the exceptional temperature stability of an mRNA based vaccine that ranges from -80°C to +70°C. While a certain range of temperatures tolerated by different rabies vaccine preparations has been reported [17–19] no vaccine format featuring a comparable temperature stability-range has been described as yet. Importantly, previous studies have shown, that conventional rabies vaccines can exhibit significantly decreased efficacy upon storage at elevated temperatures [17]. Hence, data presented here support a scenario, in which an mRNA-based rabies vaccine could circumvent the need for a cold chain, allowing vaccine transport to areas in which a rabies vaccine is needed but its supply remains a logistical challenge.

Moreover, we expect that our findings can be extended to other mRNA vaccines based on RNActive technology, given that these vaccines only differ in nucleotide sequence while the chemical nature of the active pharmaceutical ingredient, namely mRNA, remain the same. While a difference in nucleotide sequence may influence secondary structure, it seems unlikely to have any major impact on the response to thermal stress.

Thermostability contributes to an extended shelf life of vaccines even in challenging environments, and also allows for more economical stockpiling of vaccines, for example, for global preparedness against epidemic threats. Subsequent studies are needed to confirm that the exceptional temperature stability observed here can be implemented into a final recommendation for transport outside the cold chain for a licensed product in the end. Those studies will most likely need to include clinical testing and alignment of the clinical data to a potency release test.

In summary, mRNA represents a highly thermostable and efficacious vaccine platform that may contribute to simplifying global access to vaccines in the future.

## Supporting information

S1 Table Overview of efficiency of mRNA vaccine at all storage conditions assessed. Summary of induced immune responses, body weight kinetic and survival of mice after *i*.*c*. challenge infection following vaccination using vaccines stored at times and temperatures indicated.
(DOCX)
