# Supplementary material for: A thermostable messenger RNA based vaccine against rabies
Source: PLoS Negl Trop Dis. 2017 Dec 7;11(12):e0006108. doi: 10.1371/journal.pntd.0006108 (PMC5737050; doi:10.1371/journal.pntd.0006108)
Supplement: S1 Table — Summary of induced immune responses, body weight kinetic and survival of mice after i.c. challenge infection following vaccination using vaccines stored at times and temperatures indicated. (DOCX) [file pntd.0006108.s001.docx]

| **Table S1: Overview of efficiency of mRNA vaccine at all storage conditions assessed** | | | | |
| --- | --- | --- | --- | --- |
| **Storage temperature** | **Storage duration of RABV-G mRNA prior to immunization** | **VNT in IU/ml**  (median/min-max) | **Survival upon *i.c.* challenge** | **Max. mean weight loss of initial bodyweight**  (at any measured time point over 14 days after *i.c.* challenge) |
| -80°C | 6 months | 41 (14-53) | 5/5 (100%) | ≤ 10% * |
|  | 12 months | 41 (31-53) | 5/5 (100%) | ≤ 2.5% |
| 5°C | 6 months | 53 (18-70) | 5/5 (100%) | ≤ 5% |
|  | 12 months | 70 (18-70) | 5/5 (100%) | ≤ 7.5% * |
| 25°C | 6 months | 41 (14-70) | 5/5 (100%) | ≤ 5% |
|  | 12 months | 53 (23-70) | 5/5 (100%) | ≤ 2.5% |
| 40°C | 6 months | 53 (41-70) | 5/5 (100%) | ≤ 7.5% * |
|  | 1 week (reconstituted) | 61 (8-70) | 7/7 (100%) | ≤ 5% |
| 60°C | 1 month | 62 (3-70) | 8/8 (100%) | ≤ 5% |
|  | 3 months | 53 (23-57) | 8/8 (100%) | 0% |
| 70°C | 1 month | 57 (18-70) | 8/8 (100%) | ≤ 1% |
|  | 3 months | 37 (8-57) | 8/8 (100%) | 0% |
| 80°C | 3 months | 0.6 (0.1-4) | 4/8 (50%) | > 20% |
| 56°C/4°C | 20 cycles  56°C (8-9h)/4°C (15-16h) | 48 (19-57) | 8/8 (100%) | 0% |

Note: For details on individual experiments refer to main text.

* mice fully recovered within the observation time
